# Balanced splicing at the Tat-specific HIV-1 3′ss A3 is critical for HIV-1 replication

**DOI:** 10.1186/s12977-015-0154-8

**Published:** 2015-03-28

**Authors:** Steffen Erkelenz, Frank Hillebrand, Marek Widera, Stephan Theiss, Anaam Fayyaz, Daniel Degrandi, Klaus Pfeffer, Heiner Schaal

**Affiliations:** Institute of Virology, Heinrich-Heine-University Düsseldorf, D-40225 Düsseldorf, Germany; Current address: Institute of Virology, University Hospital Essen, Essen, Germany; Institute of Clinical Neuroscience and Medical Psychology, Heinrich-Heine-University Düsseldorf, Düsseldorf, Germany; Plant Biology Laboratories, Michigan State University, Michigan, USA; Institute of Medical Microbiology and Hospital Hygiene, Heinrich-Heine-University Düsseldorf, D-40225 Düsseldorf, Germany

**Keywords:** HIV-1, Tat, Viral transcription, Alternative splicing, SR proteins, SRSF2, SRSF6, Splicing regulatory element, SRE, HEXplorer

## Abstract

**Background:**

The viral regulatory protein Tat is essential for establishing a productive transcription from the 5′-LTR promoter during the early phase of viral gene expression. Formation of the Tat-encoding mRNAs requires splicing at the viral 3′ss A3, which has previously been shown to be both negatively and positively regulated by the downstream splicing regulatory elements (SREs) ESS2p and ESE2/ESS2. However, using the novel RESCUE-type computational HEXplorer algorithm, we were recently able to identify another splicing enhancer (ESE^5807-5838^, henceforth referred to as ESE_*tat*_) located between ESS2p and ESE2/ESS2. Here we show that ESE_*tat*_ has a great impact on viral *tat*-mRNA splicing and that it is fundamental for regulated 3′ss A3 usage.

**Results:**

Mutational inactivation or *locked nucleic acid* (LNA)-directed masking of the ESE_*tat*_ sequence in the context of a replication-competent virus was associated with a failure (i) to activate viral 3′ss A3 and (ii) to accumulate Tat-encoding mRNA species. Consequently, due to insufficient amounts of Tat protein efficient viral replication was drastically impaired. RNA *in vitro* binding assays revealed SRSF2 and SRSF6 as candidate splicing factors acting through ESE_*tat*_ and ESE2 for 3′ss A3 activation. This notion was supported by coexpression experiments, in which wild-type, but not ESE_*tat*_-negative provirus responded to higher levels of SRSF2 and SRSF6 proteins with higher levels of *tat*-mRNA splicing. Remarkably, we could also find that SRSF6 overexpression established an antiviral state within provirus-transfected cells, efficiently blocking virus particle production. For the anti-HIV-1 activity the arginine-serine (RS)-rich domain of the splicing factor was dispensable.

**Conclusions:**

Based on our results, we propose that splicing at 3′ss A3 is dependent on binding of the enhancing SR proteins SRSF2 and SRSF6 to the ESE_*tat*_ and ESE2 sequence. Mutational inactivation or interference specifically with ESE_*tat*_ activity by LNA-directed masking seem to account for an early stage defect in viral gene expression, probably by cutting off the supply line of Tat that HIV needs to efficiently transcribe its genome.

## Background

After integration of the human immunodeficiency *virus type 1* (HIV-1) into the host genome, the cellular RNA polymerase II (RNAPII) transcribes just one primary RNA from the 5′-long terminal repeat (5′-LTR). This pre-mRNA preserves the open reading frames (ORFs) for all of 18 different viral proteins altogether driving replication, infectivity and immune evasion [1-5]. CAP-dependent translation of almost all viral proteins predisposes the *gag*/*pol* reading frames by virtue of their proximity to the 5′ end of unspliced RNAs to be the first ones efficiently recognized by the scanning 43S ribosomal subunit. However, expression of seven other CAP-distal ORFs including Tat and Rev is indispensable for efficient viral replication. That is why a substantial amount of the primary RNA – approximately half – is diverted into the splicing pathway. Alternative splice site selection allows the excision of upstream sequences containing translational inhibitory AUGs and converts CAP-distal reading frames to CAP-proximal ones efficiently translated by the scanning ribosome.

A vast repertoire of more than 40 different viral mRNAs is processed within an infected host cell, which can be arranged by size into three major subgroups: intronless 2 kb, intron-containing 4 kb and unspliced 9 kb RNAs [6]. Among the peculiarities of viral mRNA splicing is that it constitutes a temporal gene expression profile [7,8]. Early phase of viral gene expression is characterized by the appearance of intronless 2 kb mRNAs, including Tat- and Rev-encoding transcripts. Entry into late phase of viral gene expression coincides with accumulation of Rev protein, permitting the export of intron-containing 4 kb and unspliced 9 kb mRNAs into the cytoplasm ([9], for a recent review see [10]). These would normally be retained within the cell’s nucleus, but achieve export from the nucleus via the CRM1 pathway requiring interactions between Rev and the Rev-responsive element (RRE) within the *env*-coding sequence. During the late phase of viral gene expression, the accessory and structural proteins Vif, Vpr, Vpu, and Env are translated from the respective intron-containing viral mRNAs (4 kb). Moreover, unspliced viral mRNA (9 kb) is translated into structural and enzymatic proteins or packed as genomic RNA into progeny virions.

**Figure 1:**
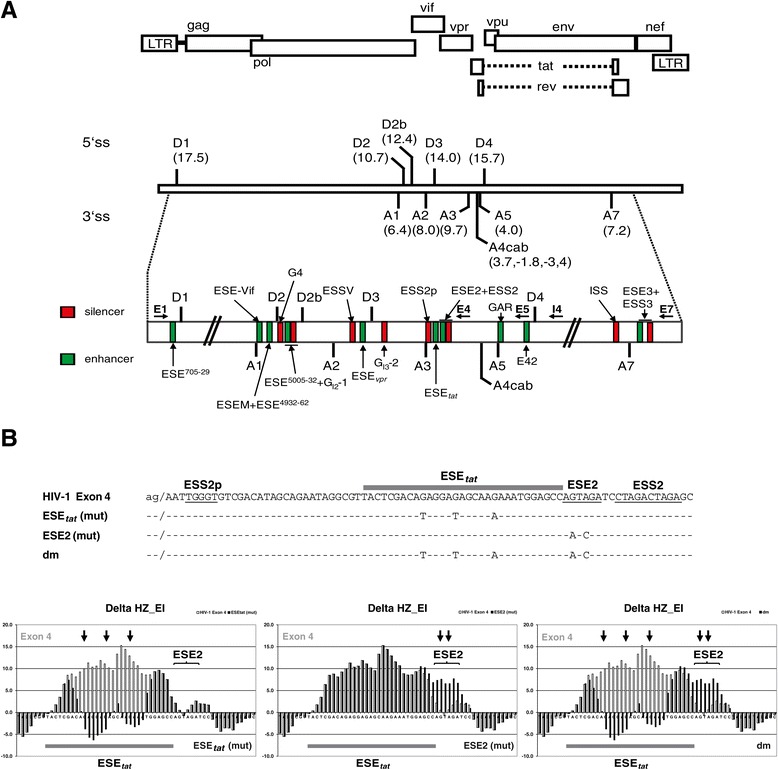
**Splicing regulatory elements (SREs) in the HIV-1 genome and mutational analysis of ESE**_tat_ **. (A)** Top: The open reading frames (ORFs) are indicated by open boxes. The long terminal repeats (LTR) are located at both ends of the provirus. Center: All HIV-1 proteins are encoded in a single primary transcript. More than 40 different viral mRNAs are produced by alternative splicing allowing efficient translation of all ORFs within the infected cell. Intrinsic strength of the 5′ss (D1 to D4) and 3′ss (A1-A7) is indicated in parentheses (5′ss: HBond Score, http://www.uni-duesseldorf.de/rna; 3′ss: MaxEntScore, http://genes.mit.edu/burgelab/maxent/Xmaxentscan_scoreseq_acc.html). Bottom: Positions of the SREs within the HIV-1 pre-mRNA: splicing enhancers (green) and silencers (red) are indicated. [ESE^705-29^ [14,40]; ESE-Vif [41]; ESEM [39]; ESE^4932-62^ [14]; ESE^5005-32^ [14]; guanosine (G) rich silencer G4 [41]; G_I2_-1 [42]; ESSV [43-45]; ESE_vpr_ [12]; G_I3_-2 [16]; ESS2p [46]; ESE2 [15,47]; ESS2 [48-50]; guanosine-adenosine rich (GAR) ESE [13,37,51]; E42 fragment [51]; ISS [52]; ESE3 [53]; ESS3 [53-55] (adapted to [51,56]). Primers used in RT-PCR analyses are indicated by arrows (forward: E1, reverse: E4, E5, I4 and E7). **(B)** Top: HIV-1 exon 4 reference (pNL4-3) and mutant sequences used in this study. The ESE_tat_ is indicated by a grey rectangle. Previously published SREs in this region are underlined. Bottom: HEXplorer score profiles for wild-type HIV-1 exon 4 reference (white) and mutant sequences (black). ESE_tat_ is indicated by a grey rectangle. ESE2 is indicated by curly braces. Positions of mutated nucleotides are indicated by arrows.

Multiple splice sites (ss) and neighboring splicing regulatory elements (SREs) present within the HIV-1 genome synergistically establish the considerable mRNA diversity (Figure 1) (for a review see [11]). Expression of each of the seven downstream ORFs is coupled to the extent of use of a specific 3′ss found in its upstream region. Splice site selection in turn is controlled by the activities of SREs in the vicinity, which can act as “on” or “off” switches for the assembly of a functional spliceosome at a given splice site, and many viral SREs have already been identified in the past (e.g. [12,13]). The identification of putative SREs has been greatly facilitated by a computational algorithm: the HEXplorer score HZ_EI_ of any given nucleotide in a specific genomic sequence depends on a symmetric neighborhood of 11 up- and downstream nucleotides. Using a RESCUE-type algorithm, HZ_EI_ is calculated as average hexamer Z-score in this neighborhood, based on hexamer overabundance in datasets of exonic compared to intronic sequences flanking constitutive human 5′ splice sites. Plotted along genomic sequences, HEXplorer score profiles reflect splice enhancing (HZ_EI_ > 0) or silencing (HZ_EI_ < 0) properties of sequence regions. For any mutation in the vicinity of a 5′ splice site, its HEXplorer score difference ΔHZ_EI_ between wild type and mutant sequences quantitatively measures the change in splice enhancing property and correlates with 5′ splice site usage [14]. Using this HEXplorer score, we were recently able to computationally identify and experimentally confirm five novel SREs being part of the viral SRE landscape [14].

In this work, we investigated the functional importance of one of these computationally identified SREs for viral replication: ESE^5807-5838^ appears to control activation of the Tat-specific 3′ss A3 and is henceforth referred to as ESE_*tat*_. Productive transcription from the viral 5′-LTR heavily relies on the expression of viral Tat protein, which facilitates transcriptional elongation by recruiting the cellular transcriptional elongation factor P-TEFb to RNAP II (for a recent review see [10]). Here we show that ESE_*tat*_ is critical for the formation of Tat-encoding mRNAs within provirus-transfected HEK293T cells and virus-infected Jurkat T-cells. ESE_*tat*_ is bound by the SR protein SRSF2, while SRSF6 binds mainly to ESE2. We provide evidence that viral replication requires an intact ESE_*tat*_, since mutational inactivation of the enhancer or locked nucleic acid-mediated masking leads to a severe defect in virus particle production, which is consistent with the defect in Tat-mRNA splicing. Notably, we also show in this study that overexpression of SRSF6 efficiently blocks the virus′ ability to replicate and that the arginine-serine (RS) rich domain is dispensable for this antiviral activity.

## Results

### The ESE_*tat*_ enhancer activity downstream of viral 3′ss A3 is necessary for *tat*-mRNA splicing in provirus-transfected cells

**Figure 2:**
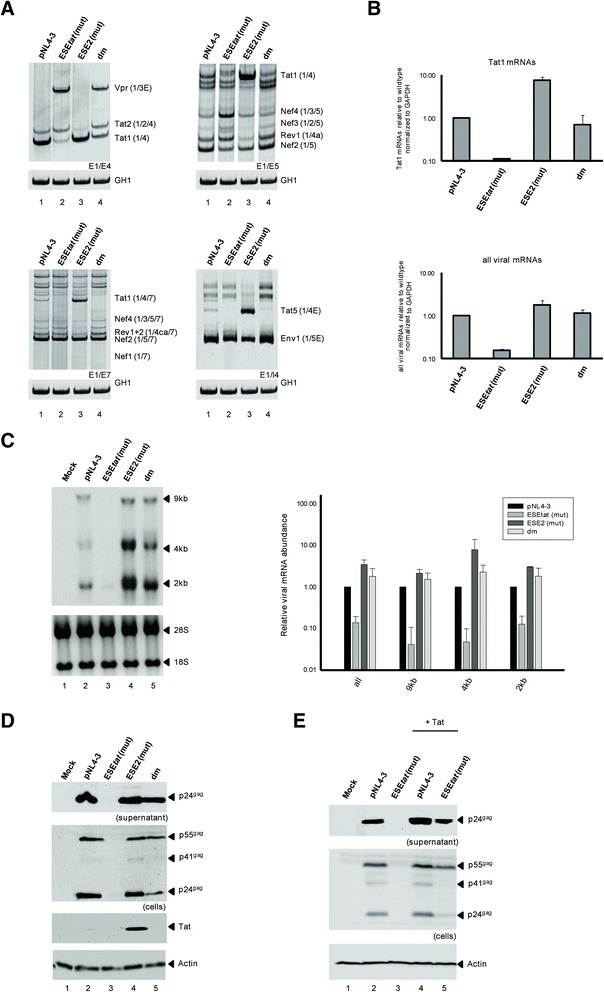
**ESE** ***tat*** **is required for activation of Tat-specific 3′ss A3. (A)** 2.5 × 105 HEK293T cells were transiently transfected with each of the proviral plasmids. 48 h post transfection, total RNA was isolated from the cells and analyzed by RT-PCR using different sets of primer pairs (primer positions are shown in Figure 1A). HIV-1 mRNA species are indicated to the right of the gel images according to the nomenclature published previously [6]. **(B)** Real-time PCR assays to specifically quantitate the relative levels of *tat*-mRNAs (top) and all viral mRNAs (bottom). For normalization we monitored the total amount of cellular GAPDH present in every sample. Data represent expression ratios relative to that of wild-type pNL4-3 (bar 1). Values and error bars show the average ± standard deviation of three independent transfection experiments. **(C)** Left: Northern blot analysis of total RNA isolated from the same RNA preparation as in **(A)**. A hybridization probe was used specifically detecting HIV-1 exon 7. Right: Quantification of Northern blot using RNAs from three independently performed transfection experiments. Data represent expression ratios relative to that of wild-type pNL4-3 (bar 1), which was set to 1. For normalization the ribosomal RNA amount of each sample was calculated. Values and error bars show the average ± standard deviation of three independent transfection experiments. **(D)** Western blot analysis of viral Gag and Tat expressed by wild-type reference and mutant provirus. Supernatants and lysates were probed with a primary antibody against HIV-1 p24gag or HIV-1 tat. Equal amounts of cell lysates were controlled by the detection of α-actin. **(E)** 2.5 × 105 HEK293T cells were transfected with 1 μg of proviral plasmids and 0.5 μg of pcDNA3.1(+) or SVctat expressing viral Tat protein from a cDNA. Western blot analysis was performed as described in **(D)**.

By applying the HEXplorer algorithm to all HIV-1 exons in order to identify splicing enhancer activities, we localized a sequence downstream of the Tat-specific 3′ss A3 with splice enhancing properties, ESE_*tat*_ (originally termed ESE^5807-5838^) [14]. Since ESE_*tat*_ lies upstream of the previously published splicing regulatory elements ESE2 and ESS2 [15] (Figure 1), we wished to analyze its role in HIV-1 pre-mRNA splicing. To this end we performed a mutational analysis and transfected HEK293T cells with pNL4-3 (GenBank Accession No. M19921) or mutant recombinant clones. The ESE_*tat*_ nucleotide substitutions were selected by the HEXplorer algorithm to maximally disrupt ESE_*tat*_ enhancing property but not the ESE2 (Figure 1B, [14]). The ESE2 mutations (Figure 1B) were selected from [15]. To monitor equal transfection efficiencies cells were cotransfected with a plasmid expressing the human growth hormone 1 (GH1). Following transfection, the HIV-1 splicing pattern was determined by semi-quantitative RT-PCR using primer pairs detecting intronless or intron-containing HIV-1 mRNA classes (for relative positions of the primers see Figure 1). In agreement with earlier studies [6,12], the expression of viral *tat*-mRNAs relative to viral *nef*- or *env*-mRNAs was rather low (Figure 2A upper right, cf. Tat1 and Nef2 or lower right, Tat5 and Env1). However, for in-depth analysis of differences in the *tat*-mRNA expression profiles of the NL4-3 variants, we additionally used the primer pair E1/E4 exclusively detecting splicing events at 3′ss A1 to A3 across the 5′-half of the viral pre-mRNA. Here we found that Tat1 was by far the most abundant isoform among all *tat*-mRNA species (Figure 2A upper left, cf. Tat1 and Tat2), which is again consistent with previous results [6,12]. As expected from the computational prediction, *tat*-mRNA levels were clearly decreased in the presence of the inactivating ESE_*tat*_ mutation (Figure 2A upper left, Tat1 cf. lane 1 and 2), indicating that the ESE_*tat*_ activity is necessary for activation of 3′ss A3. Furthermore, we repeatedly observed that reduced usage of one viral 3′ss led to activation of another [12]. Accordingly, it was found that less usage of 3′ss A3 caused higher levels of 3′ss A2 activation (Figure 2A upper left, e.g. Tat1 and Vpr cf. lane 1 and 2). However, the ESE2 mutation did not reduce but rather further increased *tat*-mRNA splicing efficiency (Figure 2A lower right, e.g. Tat5 cf. lanes 1 and 3). Although contrary to previous results [15], this finding was in agreement with the HEXplorer-based prediction, anticipating that the two inserted ESE2 point-mutations would increase rather than decrease the enhancing property of this sequence (Figure 1B). Finally, the ESE_*tat*_/ESE2 double-mutation (dm, Figure 2A upper left, lane 4) showed an intermediate *tat*-mRNA splicing phenotype compared to the respective single-mutations (cf. lanes 2 and 3 with lane 4).

Quantitative real-time PCR analyses confirmed a failure of the ESE_*tat*_ mutation to properly activate 3′ss A3, as indicated by an approximately 10-fold reduction in the levels of *tat*-mRNA (Figure 2B upper, Tat1, cf. pNL4-3 and ESE_*tat*_ (mut)). By contrast, the ESE2 mutation showed an almost 8-fold upregulation in the amount of *tat*-mRNA relative to wild-type (Figure 2B upper, Tat1, cf. pNL4-3 and ESE2 (mut)). As expected from insufficient amounts of Tat protein, which is needed for transactivation of the HIV-1 promoter, the ESE_*tat*_ mutation caused an approx. 10-fold decrease in viral RNA levels (Figure 2B lower, cf. pNL4-3 and ESE_*tat*_ (mut)). However, the ESE2 mutation showed an approx. 3-fold increase in RNA abundance (Figure 2B lower, cf. pNL4-3 and ESE2 (mut)), as it was anticipated by the higher levels of Tat-encoding viral mRNAs detected within the quantitative and semi-quantitative RT-PCR analyses. Finally, Northern blot analyses again confirmed the overall decreased and increased amounts of viral RNAs due to the ESE_*tat*_ and ESE2 mutations, respectively (Figure 2C, cf. lanes 2 – 5). Interestingly, we could detect an increased expression of spliced versus unspliced viral RNAs for the ESE2 mutant, probably because of an aberrantly high A3 splice site activation (Figure 2C, cf. lanes 2 – 4).

In Western blot analyses, inactivating the ESE_*tat*_ could also be demonstrated to impair virus particle production, as p24^gag^ levels were nearly undetectable in the supernatant of transfected cells (Figure 2D upper panel, cf. lanes 2 and 3). However, to substantiate our finding that mutation of the ESE_*tat*_ results in a failure to provide sufficient amounts of Tat protein needed to drive productive viral transcription, we wanted to examine whether splicing-independent Tat coexpression from a cDNA may rescue the ESE_*tat*_ mutation causing the replication defective phenotype. Indeed, co-transfection of HEK293T cells with pNL4-3 ESE_*tat*_ mutant and a Tat expression plasmid at least partially restored virus particle production (Figure 2E, cf. lanes 2 and 5) reinforcing the hypothesis that shortage in the accumulation of Tat protein is the major cause for the virus′ failure to successfully propagate.

### ESE_*tat*_ is critical for activation of the Tat-specific 3′ss A3 in the context of infected T-cells

**Figure 3:**
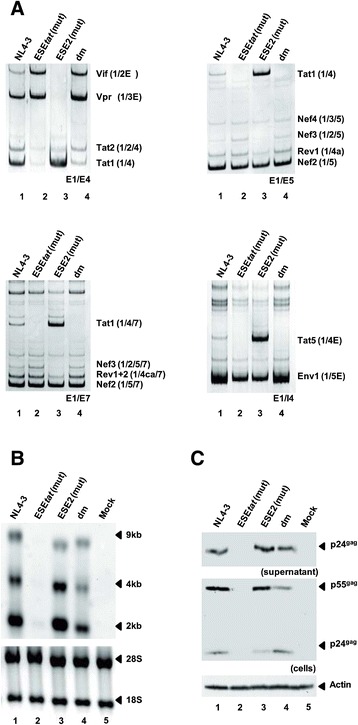
**ESE**_tat_ **-negative virus fails to efficiently replicate in T-cells. (A)** RT-PCR analysis of viral mRNAs taken from Jurkat T-cells that were infected with 10 ng p24^gag^ of wild-type or mutant NL4-3 virus. Total RNA was isolated 6 days post infection and subjected to RT-PCR analysis using different sets of primer pairs (primer positions are shown in Figure 1A). **(B)** Northern blot analysis of RNAs isolated in **(A)** using a DIG-labeled HIV-1 exon7 probe detecting all three viral mRNA species. **(C)** Western blot analysis of intracellular and supernatant HIV-1 Gag collected 6 days post infection as described above. Actin detection was used as loading control.

To extend our analyses, Jurkat T-cells were infected with equal amounts of p24 collected from the supernatants of either wild-type- or mutant provirus-transfected HEK293T cells. In agreement with the results obtained from the transfection experiments, *tat*-mRNA levels detected by RT-PCR were substantially down- or upregulated dependent on whether cells were infected with ESE_*tat*_ - or ESE2-mutated virus (Figure 3A upper right, e.g. Tat1, for ESE_*tat*_ cf. lanes 1 and 2 and for ESE2 (mut) cf. lanes 1 and 3). HIV-1 carrying both mutations produced *tat*-mRNAs at levels between those found for the respective single mutants (Figure 3A upper left, Tat1, cf. lanes 2-4). Herein, it was once again found that the absence of the ESE_*tat*_ activity could not be entirely counterbalanced by an increased ESE2 activity, since *tat*-mRNA splicing clearly failed to reach wild-type levels (Figure 3A, upper left, Tat1, cf. lanes 1 and 4).

The ESE_*tat*_-mutant derived defect in the supply of Tat protein for efficient viral transcription was recapitulated by a strong decrease of all viral RNAs detected in the Northern blot analysis (Figure 3B, cf. lanes 1 and 2). However, contrary to what was seen in the context of provirus-transfected HEK293T cells, the ESE2 mutant showed no evidence for an increased transcriptional activity in the Northern blot analyses, which might be due to a transfection-dependent higher responsiveness of the viral LTR promoter to ectopically expressed Tat (Figure 3B, cf. lanes 1 and 3). However, as a general tendency for the ESE2 mutant, we could once again observe a shift from unspliced to spliced viral RNAs hinting at higher A3 splice site activation.

Overall, the results obtained from the Northern blot analyses indicate that the maintenance of a balanced 3′ss A3 activation by ESE_*tat*_ is important for the virus’ competence to efficiently replicate. Hence, virus replication is impaired regardless of whether 3′ss A3 usage is strongly decreased (due to a lack of Tat) or increased (due to a lack of unspliced RNA) (Figure 3C, cf. lanes 1 – 3).

### Masking ESE_*tat*_ phenotypically mimics its mutational inactivation

**Figure 4:**
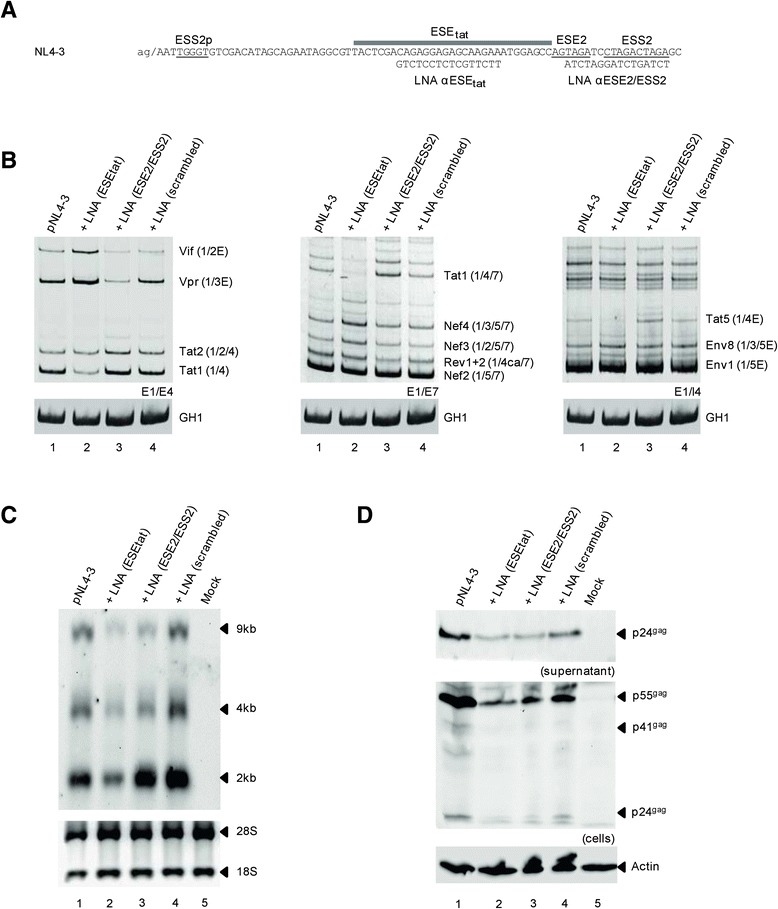
**LNA-mediated masking of ESE**_tat_ **mimics the influence of the mutated ESE**_tat_ **on the viral splicing pattern and viral particle production. (A)** Schematic illustration of the location of ESS2p, ESE_tat_, ESE2 and ESS2 as well as the binding site for the locked nucleic acids (LNAs) directed against ESE_tat_ and ESE2/ESS2 sequences. **(B)** RT-PCR analysis of viral mRNA classes. HeLa cells were transiently transfected with pNL4-3 and either ESE_tat_-LNA, the ESE2/ESS2 LNA or a scrambled LNA. Total RNA was isolated 24 h post transfection and subjected to RT-PCR analysis using different primer pairs (Figure 1A). **(C)** Northern blot analysis of total RNA collected in **(B)** using a DIG-labelled probe hybridizing to HIV-1 exon 7. **(D)** Western blot analysis of cellular and supernatant viral Gag of co-transfected cells from **(B)**. The detection of actin served as loading control.

Recently, we could inactivate the intronic HIV-1 splicing regulatory element G_I3_-2 located in intron 3 by co-transfecting HeLa cells with a locked nucleic acid (LNA) targeting the G_I3_-2 sequence [16]. To investigate whether we could also mask the exonic ESE_*tat*_ by rendering the sequence inaccessible for splicing factor binding as previously demonstrated by *in vitro* binding assays [16], we cotransfected HeLa cells with the proviral pNL4-3 plasmid and either an LNA directed against the ESE_*tat*_ sequence, the overlapping ESE2/ESS2 sequence or a randomized sequence as control (Figure 4A). In good agreement with the results obtained from the mutational analyses (cf. Figure 2), an LNA directed against the ESE_*tat*_ sequence led to a substantial reduction in the levels of Tat-encoding mRNA species detected (Figure 4B left panel, e.g. Tat1 cf. lanes 1 and 2), indicating a specific inhibition of the ESE_*tat*_ activity by sequence-directed LNA-delivery. By contrast, an LNA targeting the ESE2/ESS2 sequence revealed a shift towards Tat-encoding mRNA species (Figure 4B left panel, e.g. Vpr and Tat1 cf. lanes 1 and 3) comparable to the increased 3′ss A3 usage seen for the ESE2 mutant. However, since masking the ESE2/ESS2 sequence resulted in an even greater extent of 3′ss A3 activation (cf. Figure 2B), these results might indicate a dominant role for the negative acting ESS2 amongst both overlapping elements. As it was expected, a randomized control-oligonucleotide showed no impact on the viral splicing profile (Figure 4B left panel, e.g. Tat1 cf. lanes 1 and 4). Revisiting mutational inactivation of ESE_*tat*_, masking of the splicing enhancer sequence also led to reduced levels of viral mRNAs detected by Northern blot (Figure 4C, cf. lanes 1 and 2), while RNA levels of virus treated with the control-LNA were unaffected (Figure 4C, cf. lanes 1 and 4). Although the increase in A3 splice site usage detected after treatment with the ESE2/ESS2 LNA did not appear to be associated with a considerably higher amount of total RNA, it again led to a clear shift from unspliced towards spliced viral RNAs (Figure 4C, cf. lanes 1 and 3).

As expected from a failure of ESE_*tat*_-LNA treated virus to accumulate viral RNAs, addition of the ESE_*tat*_-specific LNA also caused a reduction in virus particle production (Figure 4D, cf. lanes 1 and 2). However, virus particle production was also affected when virus was treated with either the ESE2/ESS2- or the control-LNA, although to a much lesser extent for the latter (Figure 4D, cf. lanes 1, 3 and 4). Nevertheless, this finding suggests an additional potentially sequence-unspecific antiviral effect by LNAs, which might address viral gene expression at a step later than RNA processing and requires further investigation.

### The SR proteins SRSF2 and SRSF6 act through ESE_*tat*_ and ESE2 to activate 3′ss A3

**Figure 5:**
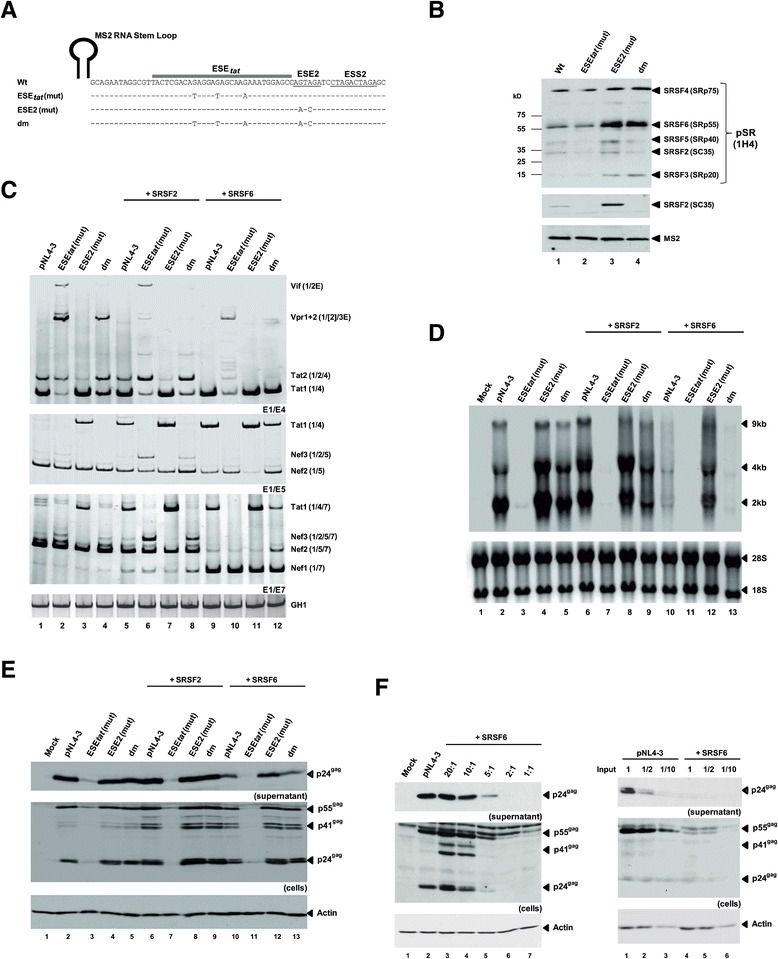
**The SR proteins SRSF2 and SRSF6 bind downstream of 3′ss A3 to control** ***tat*** **-mRNA splicing. (A)** *In vitro*-transcribed RNA substrates used for the RNA pull-down assays. Mutated nucleotides are indicated below the wild-type reference (pNL4-3) at corresponding positions and “-“ denotes wild type nucleotide. **(B)** RNAs were immobilized on Agarose beads and analyzed for the presence of SR proteins with specific antibodies directed against SRSF2 (Abcam, ab28428) or phosphorylated SR proteins (Invitrogen, 1H4G7). Recombinant MS2 coat protein was added to HeLa cell nuclear extracts and served as a control for equal precipitation efficiencies. **(C)** 2.5 × 10^5^ HEK293T were transfected with pNL4-3 or mutant provirus and pcDNA3.1(+), an SRSF2 or SRSF6-expressing plasmid. 48 h after transfection RNAs were analyzed by RT-PCR **(C)** or Northern blot **(D)**. **(E)** Cell lysates and supernatants from transfected HEK293T cells were analyzed for Gag expression as described before (see Figure 2). **(F)** Left: Cell lysates and supernatants from HEK293T cells cotransfected with pNL4-3 and gradually increasing amounts of SRSF6 expressing plasmid that were analyzed for Gag expression as described before. Right: Serial dilutions of cell lysates and supernatants from transfected HEK293T cells analyzed for Gag expression.

Previous studies suggested that activation of 3′ss A3 can be increased following coexpression of the SR protein SRSF2 and SRSF5 [17-19]. Furthermore, footprinting studies by Zahler et *al*. [15] indicated partial binding of SRSF2 not only to ESE2, but also further upstream overlapping with the HEXplorer predicted ESE_*tat*_ sequence. However, we had some additional evidence that the SR protein SRSF6 might also be involved in promoting usage of 3′ss A3. Therefore, we revisited RNA precipitation experiments upon immobilization of *in vitro* transcribed RNAs containing the wild-type and mutant sequences to identify SR proteins which in the presence of the hnRNP A1 binding site ESS2 can bind to ESE_*tat*_ or ESE2 or both (Figure 5A). To control for equal precipitation efficiencies, the 5′-end of each RNA substrate was endowed with a single copy of an RNA stem loop, serving as binding site for the bacteriophage MS2 coat protein added as recombinant protein to the nuclear extracts [20,21]. As expected, pull-down analyses revealed SRSF2 binding to the wild-type sequence (Figure 5B, upper and middle panel, lane 1). Mutating the HEXplorer predicted ESE_*tat*_ enhancer activity (Figure 1B, ESEtat (mut)) without altering the previously mapped SRSF2 binding site [15], surprisingly led to loss of SRSF2 binding, mapping an SRSF2 binding site within ESE_*tat*_ but upstream of ESE2 (Figure 5B, upper and middle panel, cf. lanes 1 and 2). Moreover, in agreement with the HEXplorer prediction (Figure 1B, ESE2 (mut) middle bar graph), levels of SRSF2 were even increased when ESE2 was mutated (Figure 5B, upper and middle panel, cf. lanes 1 and 3), suggesting that the underlying binding site is located within ESE_*tat*_. This was further supported by undetectable amounts of SRSF2 on RNAs with the ESE_*tat*_/ESE2 double mutation (dm) (Figure 5B, upper and middle panel, cf. lanes 1 and 4). Notably, the ESE_*tat*_ point mutations not only disrupted binding of SRSF2, but also binding of SRSF5 similarly described to increase *tat*-mRNA splicing upon coexpression [17,19].

Interestingly, SRSF6 binding was detected on wild-type but not reduced on ESE_*tat*_ negative RNAs (Figure 5B, upper panel, cf. lanes 1 and 2). Instead, it was significantly increased on both, ESE2 single and ESE_*tat*_/ESE2 double mutant RNAs (Figure 5B, cf. lanes 1, 3 and 4), suggesting that the underlying binding site for SRSF2 is located within ESE_*tat*_, whereas the underlying binding site for SRSF6 is within the ESE2 sequence. Furthermore, this finding indicates that the ESE2 mutations generated a new SRSF6 binding site or increased the affinity for an already existing one.

Consistently, in coexpression experiments the ESE_*tat*_ mutant showed only a poor response to higher levels of SRSF2 in comparison to the wild-type sequence (Figure 5C middle and lower panel, Tat1, cf. lanes 1and 5 with lanes 2 and 6), indicating a substantial reduction in the binding affinity of SRSF2 to the viral RNA. Surprisingly, the ESE_*tat*_ mutant also did not respond to SRSF6 upregulation (Figure 5C middle and lower panel, Tat1, cf. lanes lanes 2 and 10), although pull-down assays demonstrated that SRSF6 binding was not affected by the respective nucleotide exchanges. Given this finding, it seems that either SRSF6 is present already in saturating amounts or that SRSF6 and SRSF2 cooperatively activate Tat-specific 3′ss A3, either through two separate required mechanisms or by stabilizing each other on their respective target sequences. Since, SRSF6 binding alone is not sufficient to promote proper 3′ss A3 recognition (Figure 5C middle and lower panel, Tat1, cf. lanes 2 and 10), a cooperative effect for the splicing machinery seems to be more likely.

In agreement with this hypothesis, and given increased amounts of both bound SRSF2 and SRSF6 to the ESE2 mutant, *tat*-mRNA splicing was found to be markedly upregulated in case of the respective proviral clone coexpressed with either one of both SR proteins (Figure 5C middle and lower panel, Tat1, cf. lanes 3, 7 and 11). These results reinforced the conclusion that ESE2-affecting nucleotide changes render the target sequence more susceptible for cooperative SRSF2 and SRSF6 binding.

Finally, the double-mutant failed to respond to SRSF2 coexpression with an increase in Tat-specific 3′ss A3 activation (e.g. shift from Tat1 to Tat2 isoforms, Figure 5C upper panel, cf. lanes 4 and 8), while *tat1*-mRNA splicing could be increased in presence of higher levels of SRSF6 (e.g. shift from Tat2 to Tat1 isoform, Figure 5C upper panel, cf. lanes 4and 12). Therefore, higher local SRSF6 concentrations appear to allow promoting 3′ss A3 activation even in presence of the ESE_*tat*_ mutations, possibly by either stabilizing the weaker affinity of SRSF2 for the ESE_*tat*_ mutant sequence or by showing a higher potency to remove competing hnRNPA/B proteins from the ESS2 sequence, which were proposed to inhibit splicing of the upstream intron by directly masking the SRSF2 binding site [15]. Here it was observed that the shift towards 3′ss A3 usage upon SRSF6 overexpression was less pronounced for ESE_*tat*_/ESE2-double negative virus than for the wild-type, which indicates that an intact ESE_*tat*_ is pivotal for higher 3′ss A3 activation (Figure 5C lower panel, Tat1, cf. lanes 1 and 9 with 4 and 12).

Aside from alterations in *tat*-mRNA splicing following SR protein coexpression, SRSF6 induced additional changes within the viral splicing pattern. Accordingly, not only did SRSF6 coexpression favor *tat*-mRNA splicing (Figure 5C lower panel, Tat1, cf. lanes 1 and 9), but also exclusion of exons 4cab and 5 from Rev- and Nef-encoding mRNA species (Figure 5C lower panel, Rev1+2, Nef2 and Nef1, cf. lanes 1 and 9), which was consistent with an earlier study [22]. Thus, SRSF6 effects on viral splicing include ESE2-dependent promotion of 3′ss A3 usage, but also prevention of exons 4cab and 5 inclusion into *rev*- and *nef*-mRNAs through interference with the viral GAR element [22] or another so far unknown mechanism.

Next, we wished to determine the influence of SR protein coexpression on overall viral mRNA expression. It is striking to notice that while SRSF2 coexpression did not appear to significantly alter the levels of viral total RNAs, these were instead clearly decreased in the presence of higher SRSF6 concentrations within the cells (Figure 5D, cf. lanes 2, 6 and 10). This dramatic reduction in the overall levels of viral RNAs detected by Northern blot analyses anticipated a strong reduction in the levels of virus particles measured in the supernatant of provirus-transfected HEK293T cells with elevated SRSF6 concentrations. However, the reduction in Gag expression and virus particle production seemed to be less pronounced than expected from the Northern blot analyses (Figure 5E, cf. lanes 2, 6 and 10), which might be due to an increased Gag translational efficiency in presence of higher levels of SRSF6 [23]. To expand our analyses, and since the observed negative effect of SRSF6 on viral gene expression was in contrast to what was found in an earlier study [22], we tested varying concentrations of SRSF6 expression plasmid and could find that these inhibitory activities could already be observed at a ratio of 1:5 relative to transfected proviral plasmid (Figure 5F left panel, cf. lanes 2 and 5). To exclude loading of saturating amounts of lysate and supernatant in Figure 5E, we also measured Gag expression and virus particle production for gradual sample dilutions, reinforcing a defect in viral gene expression at higher levels of SRSF6 within provirus-transfected HEK293T cells (Figure 5F, right panel, cf. lanes 1-3 and 4-6).

Notably, among other tested splicing factors such as SRSF1 or hnRNP H, SRSF6 overexpression had the strongest negative effect on pNL4-3 gene expression (*data not shown*). We therefore conclude that higher levels of SRSF6 may predispose a target cell to a higher degree of resistance against NL4-3 replication.

### The arginine-serine (RS) rich splicing effector domain of SRSF6 is not required for the global aberrations in viral pre-mRNA splicing

**Figure 6:**
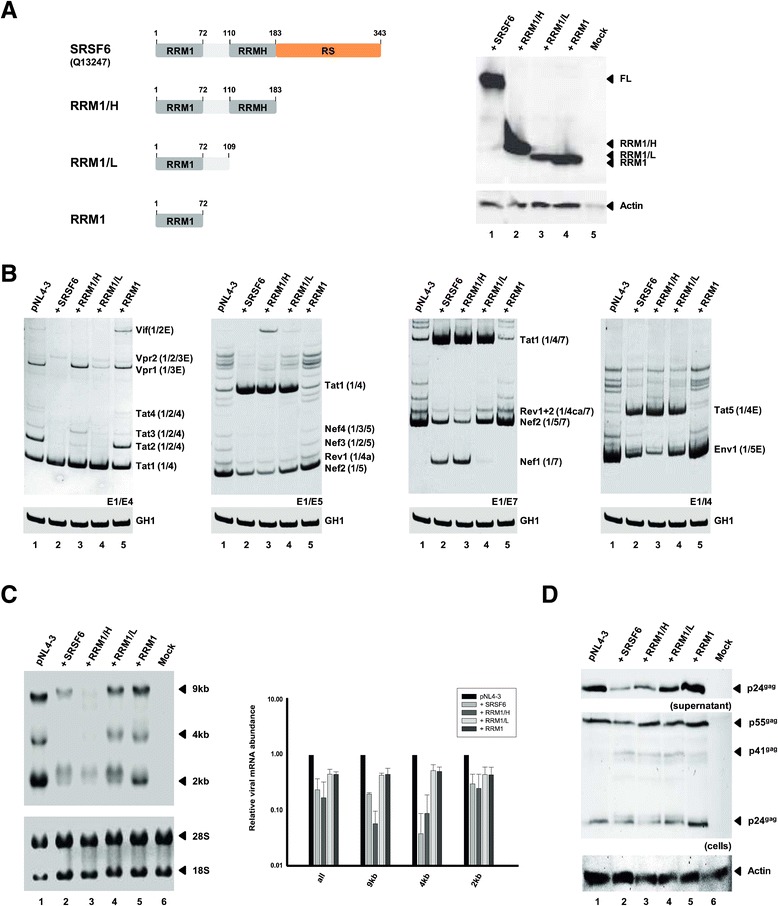
**The arginine-serine (RS) rich C-terminus as well as the RRMH domain of SRSF6 are dispensable for its antiviral activity. (A)** SRSF6 protein variants used in the coexpression experiments. Proper expression of all truncated SRSF6 variants was confirmed using an antibody specifically recognizing an HA epitope (Sigma-Aldrich, H6908), which was C-terminally fused to each mutant. HEK293T cells were transiently cotransfected with 1 μg of pNL4-3 and 1 μg of pcDNA3.1(+) or the respective SRSF6 variant-expression plasmid. Samples from the same RNA preparations were analyzed by both RT-PCR **(B)** and Northern blot **(C)**. **(D)** Cellular lysates and supernatants were analyzed by Western blot using antibodies directed against viral Gag or cellular actin (loading control). Values and error bars show the average ± standard deviation of two independent transfection experiments.

Given the unexpected finding that SRSF6 efficiently blocked virus particle production when coexpressed together with the infectious clone pNL4-3, we next wished to analyze which protein domain accounts for this antiviral activity. To this end, we generated different truncated SRSF6 isoforms whose removed regions either included only the arginine-rich (RS) domain (RRM1/H), the RS domain together with the RRMH domain (RRM1/L) or all but the RRM domain (RRM1) (Figure 6A, left panel). Proper expression of all variants was controlled by Western blotting (Figure 6A, right panel). Finally HEK293T cells were cotransfected with pNL4-3 and individually each of these SRSF6-derived variants. Again, we could observe that in the presence of higher levels of full-length SRSF6 splicing at 3′ss A3 was greatly increased (Figure 6B middle left (E1/E5), Tat1, cf. lanes 1 and 2). As a recurring theme, SRSF6 coexpression not only led to a shift in the viral splice acceptor selection from 3′ss A5 (e.g. Nef2 or Env1) towards 3′ss A3 usage (Tat1 and Tat5), but also appeared to repress inclusion of exons 4cab and 5 into viral Rev and Nef-mRNAs (Figure 6B middle right (E1/E7), Rev1+2 and Nef1, cf. lanes 1 and 2). Unexpectedly, deletion of the RS domain, which is commonly described to be the splicing effector domain of SR proteins, did not relieve SRSF6-mediated global changes in viral splicing (Figure 6B middle right (E1/E7), Tat1, Rev1+2 and Nef1, cf. lanes 1 and 3). Additional deletion of the RRMH was still not sufficient to reinduce the wild-type splicing pattern. Here, we could still detect a strong upregulation of 3′ss A3 splicing (Figure 6B middle left (E1/E5), Tat1, cf. lanes 1 and 4). However, a shift back from exon 5-lacking Nef1- to exons 4cab-including Rev1/2-mRNA and to exon 5-including Nef2-mRNA isoforms could be observed, while increased accumulation of *tat*-mRNA species due to higher 3′ss A3 activation was unaltered (Figure 6B middle left (E1/E5), Nef1 Rev1+2 or Nef2, cf. lanes 3 and 4). Given these findings, we argue that while the RRMH appears to be dispensable for 3′ss A3 activation, it appears to be necessary for inhibition of exon 5 inclusion. Surprisingly, the RS domain seems to be entirely dispensable for the ample changes within the viral pre-mRNA splicing pattern that are conferred by SRSF6.

However, when additionally the linker region was deleted and only the RRM1 of SRSF6 was expressed in pNL4-3-transfected HEK293T cells, no aberrantly increased levels of *tat*-mRNA splicing could be detected and hence, the viral splicing pattern was entirely unaffected (Figure 6B middle right (E1/E7), Tat1, cf. lanes 1 and 5). On the basis of these results, we propose that the SRSF6-mediated *tat*-mRNA oversplicing requires the 37 amino acid long linker region between RRM1 and RRMH, while both the RS domain and RRMH are dispensable. In contrast, inhibition of exons 4cab and 5 recognition by the spliceosome also requires RRMH.

The results obtained from Northern and Western blot analyses were mostly consistent with the observed activities of all tested SRSF6 variants (Figure 6C-D). Thus, it was found that coexpression of full-length SRSF6 led to a strong decrease in the levels of all viral mRNAs (Figure 6C, cf. lanes 1 and 2, ~ 5-fold) and consequently also in the levels of p24^gag^ proteins detected within the cells and supernatant (Figure 6D, cf. lanes 1 and 2). However, a total rescuing of virus RNA expression and particle production could already be achieved when the RRM1/L variant was coexpressed with proviral DNA (Figure 6C-D, cf. lanes 1, 2, and 4), indicating that the antiviral activity of SRSF6 is unrelated to the Tat oversplicing phenotype. From these results we conclude that the RRMH is a major contributor to the strong antiviral activity elicited by higher SRSF6 concentrations.

### The varying abilities of the different SRSF6 variants to localize within the nucleus correlate with their antiviral activity

**Figure 7:**
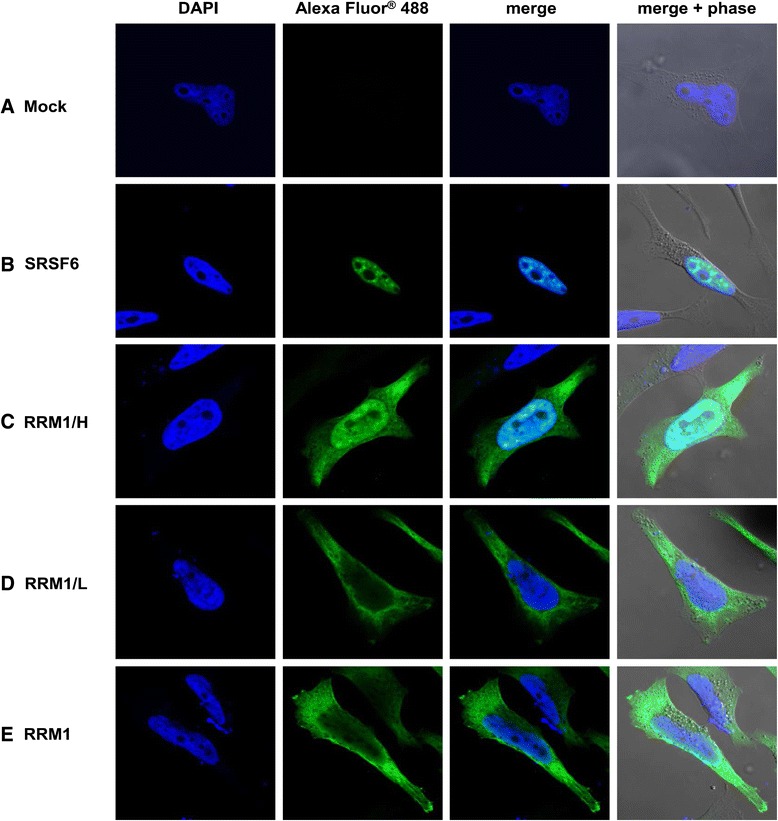
**The antiviral activity of the SRSF6 variants correlates with their subcellular localization.** Subcellular localization of the HA-tagged SRSF6 variants was unraveled by immunostaining of transiently transfected HeLa cells using an anti-HA and an anti-mouse Alexa Fluor® 488 antibody. Cell nuclei were stained with DAPI and glass slides were analyzed by confocal laser-scanning microscopy.

Next, we wished to figure out to which extent the different activities of the SRSF6 variants might be due to an altered subcellular localization. For this purpose, we transfected HeLa cells with the HA-tagged SRSF6 variants described above and analyzed the subcellular localization of these proteins by confocal laser-scanning microscopy. As expected, full length SRSF6 almost entirely accumulated within the cellular nucleus (Figure 7B) in support of the notion that the antiviral activity of SRSF6 results from changes in nuclear processing of viral or cellular mRNAs encoding for replication-relevant proteins. However, unexpectedly, the RRM1/H variant predominantly localized to the nucleus as well (Figure 7C) despite deletion of its arginine-serine (RS) domain, which was previously described to be important for nuclear targeting of SR proteins [24-26]. However, the RRM1/L and RRM1 variants both deprived of their antiviral activity were mostly found within the cytoplasm (Figure 7D and E). This may imply that SRSF6 requires a RRMH domain-dependent nuclear localization for inhibition of viral gene expression, but does not exclude a direct contribution of the RRMH domain to the SRSF6 mode of viral repression.

## Discussion

In this study, we provide evidence for the functional importance of a novel splicing enhancer – termed ESE_*tat*_ – as the dominant splicing regulatory element underlying viral Tat protein expression. Mutational disruption of this enhancer activity led to a severe defect in the formation of Tat-encoding viral mRNA species that strongly impaired virus ability to replicate within infected cells. Since LNA-directed masking of the ESE_*tat*_ sequence was also linked to a failure in the production of *tat*-mRNAs, we can exclude accidental generation of a new, artificial silencer by the inserted point-mutations.

**Figure 8:**
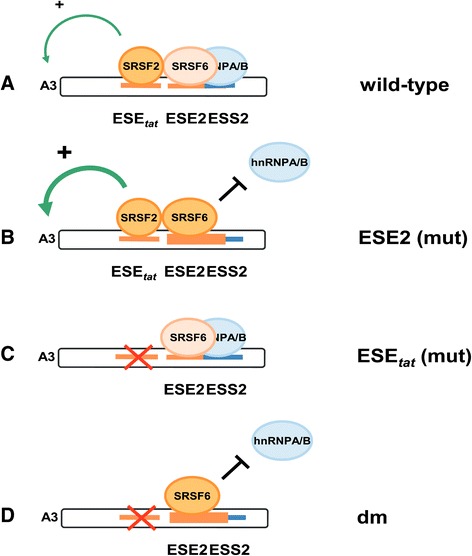
**Model for SRSF2 and SRSF6-mediated 3′ss activation. (A)** SRSF6 and hnRNP A/B proteins compete for binding to their overlapping binding sites located within ESE2 and ESS2. Depending on the SRSF6 binding efficiency **(B)**, SRSF2 interacts with the upstream ESE_tat_ and guides spliceosomal components to 3′ss A3. **(C)** Inactivation of ESE_tat_ is associated with a failure in efficient spliceosome recruitment irrespective of higher SRSF6 binding **(D)**.

Combining the results obtained from our RNA pull-down assays and those from the splicing factor coexpression experiments, we suggest that the SR proteins SRSF2 and SRSF6 act through ESE_*tat*_ and ESE2 to promote 3′ss A3 use. Both proteins are precipitated with either lower or higher efficiencies on the respective mutant RNAs (SRSF2: ESE_*tat*_; SRSF6: ESE2) than on the wild-type RNAs and higher cellular levels of SRSF2 or SRSF6 account for increased splicing of *tat*-mRNA species for ESE_*tat*_ virus but not ESE_*tat*_-negative virus. These findings are in line with several preceding studies that found a shift towards *tat*-mRNA species following upregulation of SRSF2 or SRSF6 [17-19,22,27]. We suggest a model in which neither of these two SR proteins is dispensable for splicing at 3′ss A3, but rather work together for optimized *tat*-mRNA expression. In this model, SRSF6 bound to the ESE2 sequence prevents hnRNP A/B protein recruitment to the juxtaposed ESS2, thereby stabilizing upstream SRSF2/ESE_*tat*_ interactions for activation of 3′ss A3 (Figure 8). Thus, even though SRSF6 binding alone may not be sufficient to activate 3′ss A3, SRSF6 still promotes a shift towards *tat*-mRNA splicing in the context of an intact ESE_*tat*_ when its splicing effector domain (RS domain) has been removed. Accordingly, SRSF2 seems to play a more direct and SRSF6 a more indirect role for assembly of spliceosomal components at 3′ss A3. Notably, previous studies [17,19] as well as our RNA pull-down experiments suggest an SRSF2-equivalent role for SRSF5 in ESE_*tat*_ dependent enhancement of *tat*-mRNA splicing, which needs to be clarified by upcoming studies.

Given the pivotal role of Tat for the onset of productive viral transcription, we presume that ESE_*tat*_ might be a sensor for the cell type-specific intracellular concentrations of SRSF2 and SRSF6. This is in agreement with previous results, suggesting that the lower SRSF2 expression within macrophages relative to T-cells might be the reason for suboptimal Tat expression and slower virus replication kinetics [28]. Furthermore, we could confirm our previous observation that stronger activation of one viral splice site caused weaker activation of another within the viral 3′ss selection scheme [12]. Consistent with a mutually exclusive-like splicing pattern, there was an inverse correlation between activation of Tat-specific 3′ss A3 and e.g. Vpr-specific 3′ss A2.

Although the *modus operandi* governing mutually exclusive-like splice site choice still remains unknown, we think that local constraints in delivering sufficient spliceosomal components for early splice site recognition might be causative for the observed competition among viral splice sites (for a recent review see [29]).

In particular, we could find that the splicing factor SRSF6 leads to an almost complete inhibition of viral replication when overexpressed. This was unique to SRSF6 since we could not find any comparable inhibitory effect for any other splicing factor tested so far (*data not shown*).

Revisiting the barrier functions of other human restriction factors (such as APOBEC3G), higher SRSF6 concentrations appear to bring about a cellular state that is incompatible with efficient HIV replication. Although SRSF6 does not satisfy the criteria of a *bona fide* restriction factor, at least it shares the salient feature of this protein family to dramatically impair the virus competence to successfully replicate. Given the high dependence of HIV-1 (and other retroviruses) on regulated alternative splicing, it seems plausible that splicing factors and their relative concentrations can be considered as cellular restriction factors operating in a narrow range for HIV-1 replication. A role of splicing factors in defense of foreign or selfish nucleic acids is further supported by the finding that the splicing factor hnRNP C was recently shown to protect transcriptome integrity through selective repression of cryptic splice sites within transposon-derived *Alu* elements [30]. It remains unclear by which mechanism SRSF6 inhibits HIV-1 gene expression. Based on the finding that SRSF6 overexpression appears to repress splicing of the viral Rev-specific exons 4cab, we first considered that the antiviral activity might be a result of a failure to accumulate sufficient levels of Rev protein, needed to drive the export of intron-containing and unspliced viral mRNAs to the cytoplasm ([9], for a recent review see [10]). However, although either coexpression of a Rev cDNA or use of a proviral clone bypassing Rev dependency through use of an alternative export element [31] seemed to partially overcome the SRSF6-mediated antiviral activity, neither of both was able to fully restore virus particle production (*data not shown*), indicating additional superimposed negative effects on viral gene expression.

Notably, the arginine-serine (RS)-rich domain was largely dispensable for the nuclear import, viral splicing changes and the antiviral activity of SRSF6. This is surprising, since RS domains are generally supposed to act as nuclear localization signals (NLS) and to be fundamental for the transport of SR proteins into the nucleus [24-26]. Moreover, they are considered to represent the main protein effector domain of SR proteins [20,32-34]. This may indicate that the effects of SRSF6 on viral splicing are rather based on displacement of other splicing factors from overlapping binding motifs (for instance hnRNPA/B to ESS2) [15].

However, despite an unquestioned requirement of the RRMH domain for nuclear localization, it remains elusive whether it is also directly relevant for the antiviral activity of SRSF6. This uncertainty also applies to all other protein regions (with exception of the RS domain). Discriminating the respective contributions of the different SRSF6 domains to the establishment of the antiviral state would have required equal nuclear import efficiencies conferred by fusion of a heterologous NLS. Therefore, it still awaits further studies e.g. to establish whether RRMH is directly involved in inhibition of viral gene expression or only directs efficient import of SRSF6 into the nucleus. In addition, it remains to be answered whether the failure of the RRM1 variant to affect viral mRNA splicing results from (i) inability to enter the nucleus, (ii) importance of the linker region for viral RNA substrate affinity as described for other RNA binding proteins (e.g. [35,36]) or (iii) defective protein-protein interactions. Despite some differences in data interpretation, our results are consistent with earlier studies indicating an SRSF6-associated shift within the splicing pattern towards *tat*-mRNA species [22,27] as well as a reduction in total levels of viral RNA [22,23,27]. However, in these studies primarily replication-incompetent subgenomic HIV-1 plasmids have been assayed, which might not necessarily reflect all SRSF6 coexpression-related effects such as an enhancement of viral Gag expression [23].

Some cellular splicing factors show altered expression levels during the course of an HIV-1 infection [28], encouraging the notion of splicing factors to act as barrier proteins. We suggest that higher or lower levels of SRSF6 may predispose a cell to an increased or decreased HIV-1 resistance, and that it needs to be downregulated for efficient viral replication. Therefore, profiling of splicing factor expression in different target cells may help to predict the outcome of viral replication.

## Conclusions

ESE_*tat*_ contributes to the activation of 3′ss A3 for splicing of viral *tat*-mRNAs. ESE_*tat*_-negative HIV-1 fails to sufficiently accumulate *tat*-mRNAs, resulting in a shortage of Tat trans-activator protein. Throughout coexpression experiments, we found evidence for an antiviral activity of splicing factor SRSF6, demonstrated by an almost complete loss of virus particle production. Interestingly, the arginine-serine (RS)-rich effector domain played no important role either for nuclear localization or for the negative effect on HIV-1 gene expression.

## Methods

### Oligonucleotides

Oligonucleotides were obtained from Metabion GmbH (Martinsried, Germany).

Primers used for site-directed mutagenesis (see Table 1 in separate file).

**Table 1:** **Primers used for cloning**

**Cloned construct**	**Primer**	**Sequence**
Proviral HIV-1 plasmids		
Forward		
pNL4-3 ESEtat (mut)	#4709	5′ GGG TGT CGA CAT AGC AGA ATA GGC GTT ACT CGA CAT AGG ATA GCA AAA AAT GGA GCC AGT AGA TCC TAG A3′
pNL4-3 ESE2 (mut)	#4753	5′ GGG TGT CGA CAT AGC AGA ATA GGC GTT ACT CGA CAG AGG AGA GCA AGA AAT GGA GCC AAT CGA TCC TAG ACT AGA GCC CTG G 3′
pNL4-3 ESEtat/ESE2 (mut)	#4754	5′ GGT GTC GAC ATA GCA GAA TAG GCG TTA CTC GAC ATA GGA TAG CAA AAA ATG GAG CCA ATC GAT CCT AGA CTA GAG CCC TGG 3′
Reverse	#640	5′ CAA TAC TAC TTC TTG TGG GTT GG 3′
SRSF6 expression plasmids		
Forward		
pCG-SRSF6-HA pCG-RRM1/H-HA pCG-RRM1/L-HA	#4836	5′ ATC GTA GAG CAC GCC CG 3′
pCG-RRM1-HA	#4971	5′ TCC ATA GAA GAC ACC GGG ACC 3′
Reverse		
pCG-SRSF6-HA	#5085	5′ TCT CAG GAT CCT TAC GCG TAA TCA GGA ACA TCG TAT GGG TAA CCA CCA CCA CCA TCT CTG GAA CTC GAC CTG G 3′
pCG-RRM1/H-HA	#5084	5′ TCA GGA TCC TTA CGC GTA ATC AGG AAC ATC GTA TGG GTA ACC ACC ACC ACC TGG CTT ATC TTC AAT AAG CCT AAT ATT TC 3′
pCG-RRM1/L-HA	#5083	5′ TCT CAG GAT CCT TAC GCG TAA TCA GGA ACA TCG TAT GGG TAA CCA CCA CCA CCT TCT GTA CGA ACA GGT GGT C 3′
pCG-RRM1-HA	#5086	5′ TCT CAG GAT CCT TAC GCG TAA TCA GGA ACA TCG TAT GGG TAA CCA CCA CCA CCG CCC CGG GCG TGC TCT A 3′

Primers used for semi-quantitative and quantitative RT-PCR analyses (see Table 2 in separate file).

**Table 2:** **Primers used for semi-quantitative and quantitative RT-PCR**

**Target RNA**	**Primer**	**Sequence**
Viral mRNA classes	#1544 (E1)	5′ CTT GAA AGC GAA AGT AAA GC 3′
	#3392 (E7)	5′ CGT CCC AGA TAA GTG CTA AGG 3′
	#640 (I4)	5′ CAA TAC TAC TTC TTG TGG GTT GG 3′
	#3632 (E4)	5′ TGG ATG CTT CCA GGG CTC 3′
	#3637 (E5)	5′ GAG AAG CTT GAT GAG TCT GAC 3′
Tat1	#3631	5′ CGG CGA CTG AAT TGG GTG T 3′
	#3632	5′ TGG ATG CTT CCA GGG CTC 3′
All viral RNAs	#3387	5′ TTG CTC AAT GCC ACA GCC AT 3′
	#3388	5′ TTT GAC CAC TTG CCA CCC AT 3′
GAPDH	#5163	5′ CCA CTC CTC CAC CTT TGA 3′
	#5164	5′ ACC CTG TTG CTG TAG CCA 3′

Primers used for RNA in vitro binding assays and sequences for locked nucleic acids (see Table 3 in separate file).

**Table 3:** **Primers used for RNA** ***in vitro*** **binding assays and locked nucleic acid (LNA) sequences**

**RNA substrate**	**Primer**	**Sequence**
Forward	#4759	5′ TAA TAC GAC TCA CTA TAG GG AC ATG AGG ATC ACC CAT GTG AAT TCG AAT AGG CGT TAC TCG ACA 3′
Reverse	#4761	5′ GGA TGC TTC CAG GGC 3′
LNA	Specifity	Sequence
αESE_tat_	ESE_tat_	5′ TTCTTGCTCTCCTCTG 3′
αESE2/ESS2	ESE2/ESS2	5′ TCTAGTCTAGGATCTA 3′
Scrambled	-	5′ GACGCGTCCTTACGCG 3′

### Plasmids

Proviral HIV-1 exon 4 mutants were generated by PCR mutagenesis. Therefore, the *Eco*RI/*Nde*I fragment of SV-env [37] was first replaced by the *Eco*RI/*Nde*I fragment of the proviral plasmid pNL4-3 (GenBank Accession No. M19921) to obtain preclone SV-env^5743-8892^. Subsequently, the *Sal*I/*Nde*I fragment was substituted with PCR products using appropriate forward PCR primer (see Table 1) and #640 as a reverse PCR primer containing *Sal*I and *Nde*I restriction sites. Finally, proviral HIV-1 variants were cloned by replacing the *Eco*RI/*Xho*I fragment of a pNL4-3 subclone carrying an arbitrarily choosen *Eco*RI/*Xho*I fragment of different length with mutated SV-env^5743-8892^ fragments.

SRSF6 variants were cloned by replacing the *Xma*I/*Bam*HI fragment (for SRSF6-HA; RRM1/H-HA; RRM1/L-HA) or the *Bbs*I/*Bam*HI fragment (for RRM1-HA) of pCG-SRp55 (kindly provided by E. Buratti) with PCR products using appropriate forward and reverse primers (see Table 1). After cloning, all PCR amplicons were validated by sequencing.

pXGH5 [38] was cotransfected to monitor transfection efficiencies in quantitative and semi-quantitative RT PCR analyses. The plasmid expresses the human growth hormone 1 (hGH1) under control of the mouse metallothionein-1 promoter.

### Cell culture and nucleic acid transfections

HEK 293T and HeLa cells were maintained in DMEM (Invitrogen) supplemented with 10% fetal calf serum (FCS) and 50 μg/ml of each penicillin and streptomycin (P/S) (Invitrogen). Plasmid transfections were carried out in six-well plates with 2.5×10^5^ HEK293T or HeLa cells per plate using TransIT®-LT1 reagent (Mirus Bio LLC) following the manufacturer’s instructions. LNA co-transfections were done in six-well plates with 2.5×10^5^ HeLa cells per plate grown in Opti-MEM (Invitrogen) supplemented with 5% FCS. 24 h later cells were transfected with pNL4-3 alone or pNL4-3 together with the respective LNA (80 nM) using Lipofectamine 2000 as described previously [16].

### Infection experiments

Stocks of wild-type and mutant NL4-3 virus were prepared and titers determined as described elsewhere [16]. 5×10^5^ Jurkat T-cells were infected with 10 ng of p24^gag^ of wild-type and mutant virus in serum-free RPMI medium. 6 h later infected cells were washed in PBS (Invitrogen) and resuspended in 10% FCS and 1% P/S-containing RPMI medium. 6 days post infection cells and supernatants were collected to obtain RNA and protein samples for further analyses.

### RNA extraction, RT-PCR and Northern blot analyses

Total RNA samples were collected 48 h post transfection or 6 days post infection. For RT-PCR analyses RNA was reverse transcribed using Superscript III Reverse Transcriptase (Invitrogen) and Oligo(dT) primer (Invitrogen). For semi-quantitative analyses of viral *tat* mRNAs and *vpr* mRNA splicing, cDNA was used in a PCR reaction with primers #1544 (E1) and #3632 (E4) (see Table 2). For the analysis of intronless 2 kb HIV-1 mRNAs, a PCR reaction was carried out with forward primer #1544 (E1) and reverse primer #3392 (E7). Intron -containing 4 kb HIV-1 mRNAs were detected with primers #1544 (E1) and #640 (I4). Finally, for improved resolution of RT-PCR products derived from central splice acceptor selection another PCR reaction was performed with primers #1544 (E1) and #3637 (E5). A separate PCR reaction with primer pair #1224/#1225 detecting GH1-mRNA was carried out to monitor for equal transfection efficiencies. All GH1-mRNA RT-PCR reactions were performed at 26 cycles within the linear range of amplification.

All primer sequences used for semi-quantitative RT-PCR analyses are listed in Table 2. PCR products were separated on 8% non-denaturing polyacrylamide gels and stained with ethidium bromide for visualization. Real-time PCR assays for the quantification of single mRNA species were carried out with primer pair #3631/#3632 for Tat1-mRNA and #3387/#3388 for overall viral mRNA levels. For normalization, primers #5163 and #5164 were used monitoring cellular GAPDH expression present in each sample. Fluorescence emission was read by a Light Cycler 1.5 (Roche). Data are presented as the average of three independently performed RT-PCR experiments.

For Northern blot analysis of viral mRNAs, total RNA was separated on denaturating 1% agarose gels, capillary blotted onto positively charged nylon membranes and probed with an digoxigenin (DIG)-labeled HIV-1 exon 7 PCR product (#3387/#3388).

### Antibodies

The following primary antibodies were used for immunoblot analysis: mouse antibody against α-actin (A2228) and rabbit antibody against HA (H6908) were both obtained from Sigma-Aldrich. Sheep antibody against HIV-1 p24 was purchased from Biochrom AG. Rabbit antibodies directed against viral Tat (ab43014) and SRSF2 (ab28428) were provided by Abcam. Mouse antibody against phosphorylated SR proteins (1H4G7) was obtained from Invitrogen. Rabbit antibody against MS2 was provided by Tetracore (TC7004). For detection, we used a horseradish peroxidase (HRP)-conjugated anti-mouse antibody (NA931) from GE Healthcare, a HRP-conjugated anti-rabbit antibody (A6154) from Sigma-Aldrich and a HRP-conjugated anti-sheep antibody from Jackson Immunoresearch Laboratories Inc. For immunofluorescence analysis we used an anti-HA antibody (Sigma-Aldrich, clone HA-7) to detect the HA-tagged SRSF6 variants and an Alexa Fluor® 488 conjugated AffiniPure goat anti-mouse IgG (1:200; Jackson ImmunoResearch) as secondary antibody.

### Protein analysis

Transfected cells were lysed in RIPA buffer (25 mM Tris·HCl pH 7.6, 150 mM NaCl, 1% NP-40, 1% sodium deoxycholate, 0.1% SDS, protease inhibitor cocktail (Roche)). Proteins were separated by SDS polyacrylamide gel electrophoresis, transferred to nitrocellulose membrane and subjected to immunoblotting procedure. Membranes were incubated with the respective primary and secondary antibodies and developed with ECL chemiluminescence reagents (GE Healthcare).

### Immobilization of RNA on agarose beads and RNA affinity assays

For *in vitro* transcription of wild-type and mutant substrate RNAs, DNA templates were amplified from respective proviral plasmids with forward primer #4759 containing a T7 promoter sequence and a single copy of an MS2 RNA binding site at the 5′-end and #4761 as a reverse primer (see Table 3). RNA was synthesized using the RiboMax™ large scale RNA production system (P1300, Promega) according to the manufacturer’s instructions. Substrate RNAs were covalently coupled to adipidic acid dihydrazide-Agarose beads as previously described [12,20,39]. Subsequently, immobilized RNAs were incubated in 30% HeLa cell nuclear extract (Cilbiotech)/buffer D (20 mM HEPES-KOH [pH 7.9], 5% glycerol, 0.1 M KCl, 0.2 mM EDTA, 0.5 mM DTT) for 20 min at 30°C. Recombinant MS2 coat protein was added to nuclear extract dilutions to monitor equal precipitation efficiencies. After washing off unspecifically bound proteins, remaining fraction on the RNAs was eluted by addition of an equal volume of 2× protein sample buffer and heating at 95°C for 10 min. Samples were then resolved by SDS-PAGE and transferred to nitrocellulose membranes for probing with specific antibodies.

### Immunofluorescence analyses

HeLa cells were transiently transfected with plasmids expressing the HA-tagged SRSF6 variants (SRSF6-HA; RRM1/H-HA; RRM1/L-HA and RRM1-HA). 24 hrs post transfection cells were fixed with 4% paraformaldehyde (wt/vol) in PBS (10 min, RT), permeabilized with 0.3% Triton X-100 (vol/vol) in PBS (10 min, RT) and blocked with 2% (vol/vol) normal goat serum (DaKoCytomation) for 30 min. For staining of the overexpressed SRSF6 variants the cells were incubated with a monoclonal mouse anti-HA antibody at a concentration of 1:750 diluted in PBS with 0.2% normal goat serum. After 1 h the antibody-solution was removed, cells were washed three times with PBS for 5 minutes and subsequently incubated with Alexa Fluor® 488 conjugated AffiniPure goat anti-mouse IgG diluted in PBS with 0.2% normal goat serum for 45 min. This was followed by two times washing of the cells with PBS, the staining of the nuclei with DAPI (Invitrogen) (1:5000 in PBS) for 3 min and the fixing of the cover slips on glass slides with FluoromountG (Southern Biotech). The localization of the SRSF6 variants was then analyzed using a LSM780 confocal microscope (Zeiss, Oberkochen, Germany). To avoid crosstalk in the detection of the used fluorophores, multitracking scanning mode was used. Image analyses and processing was performed with ZEN (Zeiss).

## Abbreviations

SRE: Splicing regulatory element
HIV: Human immunodeficiency virus
SRSF: SR splicing factor
ORF: Open reading frame
ELISA: Enzyme-linked immunosorbent assay
LNA: Locked nucleic acid
HBS: HBond score

## References

[1] Frankel AD, Young JA (1998). HIV-1: fifteen proteins and an RNA. Annu Rev Biochem.

[2] Jager S, Cimermancic P, Gulbahce N, Johnson JR, McGovern KE, Clarke SC (2012). Global landscape of HIV-human protein complexes. Nature.

[3] Gao F, Bonsignori M, Liao HX, Kumar A, Xia SM, Lu X (2014). Cooperation of B cell lineages in induction of HIV-1-broadly neutralizing antibodies. Cell.

[4] Kwong PD, Mascola JR, Nabel GJ (2013). Broadly neutralizing antibodies and the search for an HIV-1 vaccine: the end of the beginning. Nat Rev Immunol.

[5] Scheid JF, Mouquet H, Feldhahn N, Seaman MS, Velinzon K, Pietzsch J (2009). Broad diversity of neutralizing antibodies isolated from memory B cells in HIV-infected individuals. Nature.

[6] Purcell DF, Martin MA (1993). Alternative splicing of human immunodeficiency virus type 1 mRNA modulates viral protein expression, replication, and infectivity. J Virol.

[7] Klotman ME, Kim S, Buchbinder A, DeRossi A, Baltimore D, Wong-Staal F (1991). Kinetics of expression of multiply spliced RNA in early human immunodeficiency virus type 1 infection of lymphocytes and monocytes. Proc Natl Acad Sci U S A.

[8] Kim SY, Byrn R, Groopman J, Baltimore D (1989). Temporal aspects of DNA and RNA synthesis during human immunodeficiency virus infection: evidence for differential gene expression. J Virol.

[9] Cullen BR (1991). Regulation of human immunodeficiency virus replication. Annu Rev Microbiol.

[10] Karn J, Stoltzfus CM (2012). Transcriptional and posttranscriptional regulation of HIV-1 gene expression. Cold Spring Harbor Perspect Med.

[11] Stoltzfus CM (2009). Chapter 1. Regulation of HIV-1 alternative RNA splicing and its role in virus replication. Adv Virus Res.

[12] Erkelenz S, Poschmann G, Theiss S, Stefanski A, Hillebrand F, Otte M (2013). Tra2-mediated recognition of HIV-1 5′ splice site D3 as a key factor in the processing of vpr mRNA. J Virol.

[13] Caputi M, Freund M, Kammler S, Asang C, Schaal H (2004). A bidirectional SF2/ASF- and SRp40-dependent splicing enhancer regulates human immunodeficiency virus type 1 rev, env, vpu, and nef gene expression. J Virol.

[14] Erkelenz S, Theiss S, Otte M, Widera M, Peter JO, Schaal H (2014). Genomic HEXploring allows landscaping of novel potential splicing regulatory elements. Nucleic Acids Res.

[15] Zahler AM, Damgaard CK, Kjems J, Caputi M (2004). SC35 and heterogeneous nuclear ribonucleoprotein A/B proteins bind to a juxtaposed exonic splicing enhancer/exonic splicing silencer element to regulate HIV-1 tat exon 2 splicing. J Biol Chem.

[16] Widera M, Hillebrand F, Erkelenz S, Vasudevan A, Munk C, Schaal H (2014). A functional conserved intronic G run in HIV-1 intron 3 is critical to counteract APOBEC3G-mediated host restriction. Retrovirology.

[17] Jablonski JA, Caputi M (2009). Role of cellular RNA processing factors in human immunodeficiency virus type 1 mRNA metabolism, replication, and infectivity. J Virol.

[18] Jacquenet S, Decimo D, Muriaux D, Darlix JL (2005). Dual effect of the SR proteins ASF/SF2, SC35 and 9G8 on HIV-1 RNA splicing and virion production. Retrovirology.

[19] Ropers D, Ayadi L, Gattoni R, Jacquenet S, Damier L, Branlant C (2004). Differential effects of the SR proteins 9G8, SC35, ASF/SF2, and SRp40 on the utilization of the A1 to A5 splicing sites of HIV-1 RNA. J Biol Chem.

[20] Erkelenz S, Mueller WF, Evans MS, Busch A, Schoneweis K, Hertel KJ (2013). Position-dependent splicing activation and repression by SR and hnRNP proteins rely on common mechanisms. RNA.

[21] Singh KK, Erkelenz S, Rattay S, Dehof AK, Hildebrandt A, Schulze-Osthoff K (2010). Human SAP18 mediates assembly of a splicing regulatory multiprotein complex via its ubiquitin-like fold. RNA.

[22] Tranell A, Tingsborg S, Fenyo EM, Schwartz S (2011). Inhibition of splicing by serine-arginine rich protein 55 (SRp55) causes the appearance of partially spliced HIV-1 mRNAs in the cytoplasm. Virus Res.

[23] Swanson CM, Sherer NM, Malim MH (2010). SRp40 and SRp55 promote the translation of unspliced human immunodeficiency virus type 1 RNA. J Virol.

[24] Caceres JF, Misteli T, Screaton GR, Spector DL, Krainer AR (1997). Role of the modular domains of SR proteins in subnuclear localization and alternative splicing specificity. J Cell Biol.

[25] Lai MC, Lin RI, Huang SY, Tsai CW, Tarn WY (2000). A human importin-beta family protein, transportin-SR2, interacts with the phosphorylated RS domain of SR proteins. J Biol Chem.

[26] Kataoka N, Bachorik JL, Dreyfuss G (1999). Transportin-SR, a nuclear import receptor for SR proteins. J Cell Biol.

[27] Tranell A, Fenyo EM, Schwartz S (2010). Serine- and arginine-rich proteins 55 and 75 (SRp55 and SRp75) induce production of HIV-1 vpr mRNA by inhibiting the 5′-splice site of exon 3. J Biol Chem.

[28] Dowling D, Nasr-Esfahani S, Tan CH, O‘Brien K, Howard JL, Jans DA (2008). HIV-1 infection induces changes in expression of cellular splicing factors that regulate alternative viral splicing and virus production in macrophages. Retrovirology.

[29] Roca X, Krainer AR, Eperon IC (2013). Pick one, but be quick: 5′ splice sites and the problems of too many choices. Genes Dev.

[30] Zarnack K, Konig J, Tajnik M, Martincorena I, Eustermann S, Stevant I (2013). Direct competition between hnRNP C and U2AF65 protects the transcriptome from the exonization of Alu elements. Cell.

[31] Smulevitch S, Bear J, Alicea C, Rosati M, Jalah R, Zolotukhin AS (2006). RTE and CTE mRNA export elements synergistically increase expression of unstable, Rev-dependent HIV SIV mRNAs. Retrovirology.

[32] Kohtz JD, Jamison SF, Will CL, Zuo P, Luhrmann R, Garcia-Blanco MA (1994). Protein-protein interactions and 5′-splice-site recognition in mammalian mRNA precursors. Nature.

[33] Fu XD, Maniatis T (1992). The 35-kDa mammalian splicing factor SC35 mediates specific interactions between U1 and U2 small nuclear ribonucleoprotein particles at the 3′ splice site. Proc Natl Acad Sci U S A.

[34] Wu JY, Maniatis T (1993). Specific interactions between proteins implicated in splice site selection and regulated alternative splicing. Cell.

[35] Fialcowitz-White EJ, Brewer BY, Ballin JD, Willis CD, Toth EA, Wilson GM (2007). Specific protein domains mediate cooperative assembly of HuR oligomers on AU-rich mRNA-destabilizing sequences. J Biol Chem.

[36] Kim HS, Headey SJ, Yoga YM, Scanlon MJ, Gorospe M, Wilce MC (2013). Distinct binding properties of TIAR RRMs and linker region. RNA Biol.

[37] Kammler S, Leurs C, Freund M, Krummheuer J, Seidel K, Tange TO (2001). The sequence complementarity between HIV-1 5′ splice site SD4 and U1 snRNA determines the steady-state level of an unstable env pre-mRNA. RNA.

[38] Selden RF, Howie KB, Rowe ME, Goodman HM, Moore DD (1986). Human growth hormone as a reporter gene in regulation studies employing transient gene expression. Mol Cell Biol.

[39] Kammler S, Otte M, Hauber I, Kjems J, Hauber J, Schaal H (2006). The strength of the HIV-1 3′ splice sites affects Rev function. Retrovirology.

[40] Asang C, Erkelenz S, Schaal H (2012). The HIV-1 major splice donor D1 is activated by splicing enhancer elements within the leader region and the p17-inhibitory sequence. Virology.

[41] Exline CM, Feng Z, Stoltzfus CM (2008). Negative and positive mRNA splicing elements act competitively to regulate human immunodeficiency virus type 1 vif gene expression. J Virol.

[42] Widera M, Erkelenz S, Hillebrand F, Krikoni A, Widera D, Kaisers W (2013). An Intronic G run within HIV-1 intron 2 is critical for splicing regulation of vif mRNA. J Virol.

[43] Madsen JM, Stoltzfus CM (2005). An exonic splicing silencer downstream of the 3′ splice site A2 is required for efficient human immunodeficiency virus type 1 replication. J Virol.

[44] Domsic JK, Wang Y, Mayeda A, Krainer AR, Stoltzfus CM (2003). Human immunodeficiency virus type 1 hnRNP A/B-dependent exonic splicing silencer ESSV antagonizes binding of U2AF65 to viral polypyrimidine tracts. Mol Cell Biol.

[45] Bilodeau PS, Domsic JK, Mayeda A, Krainer AR, Stoltzfus CM (2001). RNA splicing at human immunodeficiency virus type 1 3′ splice site A2 is regulated by binding of hnRNP A/B proteins to an exonic splicing silencer element. J Virol.

[46] Jacquenet S, Mereau A, Bilodeau PS, Damier L, Stoltzfus CM, Branlant C (2001). A second exon splicing silencer within human immunodeficiency virus type 1 tat exon 2 represses splicing of Tat mRNA and binds protein hnRNP H. J Biol Chem.

[47] Hallay H, Locker N, Ayadi L, Ropers D, Guittet E, Branlant C (2006). Biochemical and NMR study on the competition between proteins SC35, SRp40, and heterogeneous nuclear ribonucleoprotein A1 at the HIV-1 Tat exon 2 splicing site. J Biol Chem.

[48] Si Z, Amendt BA, Stoltzfus CM (1997). Splicing efficiency of human immunodeficiency virus type 1 tat RNA is determined by both a suboptimal 3′ splice site and a 10 nucleotide exon splicing silencer element located within tat exon 2. Nucleic Acids Res.

[49] Amendt BA, Hesslein D, Chang LJ, Stoltzfus CM (1994). Presence of negative and positive cis-acting RNA splicing elements within and flanking the first tat coding exon of human immunodeficiency virus type 1. Mol Cell Biol.

[50] Caputi M, Mayeda A, Krainer AR, Zahler AM (1999). hnRNP A/B proteins are required for inhibition of HIV-1 pre-mRNA splicing. EMBO J.

[51] Asang C, Hauber I, Schaal H (2008). Insights into the selective activation of alternatively used splice acceptors by the human immunodeficiency virus type-1 bidirectional splicing enhancer. Nucleic Acids Res.

[52] Tange TO, Damgaard CK, Guth S, Valcarcel J, Kjems J (2001). The hnRNP A1 protein regulates HIV-1 tat splicing via a novel intron silencer element. EMBO J.

[53] Staffa A, Cochrane A (1995). Identification of positive and negative splicing regulatory elements within the terminal tat-rev exon of human immunodeficiency virus type 1. Mol Cell Biol.

[54] Amendt BA, Si ZH, Stoltzfus CM (1995). Presence of exon splicing silencers within human immunodeficiency virus type 1 tat exon 2 and tat-rev exon 3: evidence for inhibition mediated by cellular factors. Mol Cell Biol.

[55] Si ZH, Rauch D, Stoltzfus CM (1998). The exon splicing silencer in human immunodeficiency virus type 1 Tat exon 3 is bipartite and acts early in spliceosome assembly. Mol Cell Biol.

[56] Stoltzfus CM, Madsen JM (2006). Role of viral splicing elements and cellular RNA binding proteins in regulation of HIV-1 alternative RNA splicing. Curr HIV Res.

